# Serosurveillance of SARS-CoV-2 in Welsh Blood Donors: Establishment of the surveillance system and results up to November 2022

**DOI:** 10.2807/1560-7917.ES.2023.28.19.2200473

**Published:** 2023-05-11

**Authors:** Sophie Harker, Siân Elizabeth James, James Murphy, Ben Davies, Catherine Moore, Brian P Tennant, John Geen, Daniel Thomas

**Affiliations:** 1 Communicable Diseases Surveillance Centre, Public Health Wales, Cardiff, United Kingdom; 2 Research Development and Innovation, Welsh Blood Service, Pontyclun, United Kingdom; 3 Laboratory Medicine, Swansea Bay University Health Board, Swansea, United Kingdom; 4 Wales Specialist Virology Centre, Public Health Wales, Cardiff, United Kingdom; 5 Clinical Biochemistry Service, Cwm Taf Morgannwg University Health Board, Llantrisant, United Kingdom

**Keywords:** SARS-CoV-2, epidemiology, surveillance, serology

## Abstract

**Background:**

In 2020, Wales experienced some of the highest rates of confirmed COVID-19 cases in Europe. We set up a serosurveillance scheme using residual samples from blood donations to inform the pandemic response in Wales.

**Aim:**

To identify changes in SARS-CoV-2 antibody seroprevalence in Wales by time, demography and location.

**Methods:**

Residual samples from blood donations made in Wales between 29 June 2020 and 20 November 2022 were tested for antibodies to the nucleocapsid antigen (anti-N) of SARS-CoV-2, resulting from natural infection. Donations made between 12 April 2021 and 20 November 2022 were also tested for antibodies to the spike antigen (anti-S) occurring as a result of natural infection and vaccination.

**Results:**

Age-standardised seroprevalence of anti-N antibodies in donors remained stable (4.4–5.5%) until November 2020 before increasing to 16.7% by February 2021. Trends remained steady until November 2021 before increasing, peaking in November 2022 (80.2%). For anti-S, seroprevalence increased from 67.1% to 98.6% between May and September 2021, then remained above 99%. Anti-N seroprevalence was highest in younger donors and in donors living in urban South Wales. In contrast, seroprevalence of anti-S was highest in older donors and was similar across regions. No significant difference was observed by sex. Seroprevalence of anti-N antibodies was higher in Black, Asian and other minority ethnicities (self-reported) compared with White donors, with the converse observed for anti-S antibodies.

**Conclusion:**

We successfully set up long-term serological surveillance of SARS-CoV-2 using residual samples from blood donations, demonstrating variation based on age, ethnicity and location.

**Key public health message**

**What did you want to address in this study?**

Serosurveillance can provide vital information about the circulation of disease in a population as a whole, including those who may have experienced mild or asymptomatic disease. Our surveillance system aimed to monitor the SARS-CoV-2 antibody responses of the Welsh blood donor population over time, in order to identify trends in natural exposure to the virus and vaccination.

**What have we learnt from this study?**

The proportion of donors with vaccine-mediated (anti-S) antibodies increased over time with the majority having protection by the end of the study. The proportion of donors who had experienced a natural infection (indicated by anti-N) reached 80% by November 2022, suggesting high levels of disease transmission. Age, ethnicity and location of residence have a significant impact on the presence of antibodies, however sex does not.

**What are the implications of your findings for public health?**

Serosurveillance is an effective means of monitoring exposure to SARS-CoV-2 within a population. Demographic variations have a significant impact on SARS-CoV-2 antibody prevalence. Understanding differences in immunity across a region/country allows for targeted public health action.

## Introduction

The United Kingdom (UK) experienced some of the highest cumulative coronavirus disease (COVID-19) case numbers [1] and deaths [2] in Europe during the first year of the pandemic. In Wales, numbers of clinical cases and fatalities increased rapidly throughout early 2020, with the daily average infection rate becoming the second highest in Europe by October 2020 (1,054 cases per million [3]). During successive waves, transmission remained persistently high, particularly in some of the post-industrial communities of South Wales [4]. This has been despite a highly successful immunisation programme, implemented by the National Health Service in Wales. Vaccination coverage in the adult population was one of the highest in Europe by the end of 2021 [5] which led to a change in the epidemiology of COVID-19, where case-hospitalisation and case-fatality ratios reduced significantly [4].

Surveillance of COVID-19 in Wales was carried out, primarily by following trends in: people who had a positive SARS-CoV-2 PCR (PCR) test result; people hospitalised with COVID-19; and people who died within 28 days of a confirmed PCR test. Changes in testing policy during the epidemic in Wales led to bias in case counts and rates, two of the key surveillance metrics. Furthermore, hospitalisation and mortality trends only included those with severe clinical features, excluding those with mild or asymptomatic infection.

Serological surveillance for antibodies has been shown to be an effective method of monitoring the spread of infection in a population [6-10]. Serosurveillance can provide vital information about the circulation of disease in a population as a whole, including those who may have experienced mild or asymptomatic disease. Additionally, testing can detect vaccine-mediated antibody responses, giving an overall indication of susceptibility to the SARS-CoV-2 virus in a community.

Throughout the pandemic, the Welsh Blood Service (WBS) has continued to collect blood donations from volunteer donors. Blood donors are a convenient sample population for serological surveillance as they are accessible and provide sampling opportunities as part of blood product retrieval. In order to provide information on community exposure to SARS-CoV-2 to inform the pandemic response in Wales, we set up a serosurveillance system using residual samples from donated blood. We describe the setting up of the serosurveillance project and present data on the seroprevalence of SARS-CoV-2 antibody in Welsh blood donors, by time, demography and location.

## Methods

### Design and establishment of the surveillance system

The establishment of this surveillance system was a collaboration between WBS, Swansea Bay University Health Board (SBUHB) and Public Health Wales (PHW), beginning in June 2020, with Cwm Taf Morgannwg University Health Board (CTMUHB) joining in January 2022. A steering group, comprising of members from each organisation, met regularly throughout the project. The system was composed of three stages: (i) sampling; (ii) testing; (iii) data analysis and feedback.

### Sampling population

The population comprised volunteer donors aged 17 and over, donating whole blood between 29 June 2020 and 20 November 2022, to WBS, part of the National Health Service in Wales. Quota sampling was used with an aim of including the first one hundred samples each day. Demographic and clinical information relating to the donor and donation were recorded. The demographic information provided to PHW was not personally identifiable, however a donor key was supplied to identify repeat donations from an individual. Donor characteristics provided were age, sex, ethnicity and area of residence (Lower Layer Super Output Areas (LSOA)). Initially, sex was defined as self-reported current gender. However, in 2022, WBS updated this to sex assigned at birth. All blood donors were asked to self-report their ethnicity, with 3% responding ‘no stated ethnicity’.

Samples were waste products from blood manufacturing that would normally be discarded. Room temperature samples were delivered to SBUHB or CTMUHB by courier on a daily basis with an electronic list of samples included.

### Antibody testing and assay verification

Antibody testing was carried out at the biochemistry laboratories at SBUHB and CTMUHB. Initially, samples were only tested for antibodies to the nucleocapsid antigen (anti-N, resulting from natural infection). However, donations made after 12 April 2021 were also tested for antibodies to the spike antigen (anti-S, natural infection and/or vaccine-mediated antibodies). Samples negative for anti-N and anti-S antibodies usually indicate the absence of recent SARS-CoV-2 infection or vaccination; those positive for anti-N and anti-S antibodies indicate recent natural infection; and those positive for anti-S antibodies only indicate recent vaccination without infection. Testing was carried out using the Roche Immunoassay (Roche e801 and e602; Hoffmann-La Roche Ltd, Burgess Hill, United Kingdom). Results were determined by employing electrochemiluminescence as the output signal with a positive cut-off index (COI) of 1.0 for the anti-N antibodies and 0.8 U/mL for anti-S antibodies. 

Local verifications of the antibody assays were performed on the e801 platform. Precision was assessed by performing replicate measurements on positive and negative patient pools across 5 days (n = 25). The anti-N within-run precision coefficient of variation (CV) was 1.9% at 0.10 COI and 1.7% at 13.5 COI; between-batch CV was 3.6% at 0.10 COI and 2.3% at 13.5 COI. The anti-S within-run CV was 5.6% at 0.55 U/mL and 2.8% at 44.7 U/mL; between-batch CV was 7.2% at 0.55 U/mL and 2.9% at 44.7 U/mL.

Assay specificity (true negativity) was assessed by analysing anonymised samples stored from 2019 (pre-pandemic), with anti-n = 100% (95% CI: 92–100, n = 58) and anti-S = 100% (95% CI: 89–100, n = 40). Sensitivity (true positivity), was assessed by analysing samples collected from donors > 14 days after a positive SARS-CoV-2 PCR result or vaccination (anti-S only), with anti-n = 94% (95% CI: 88–98%, n = 124) and anti-S = 100% (95% CI: 93–100%, n = 61).

Pre- and post-vaccination samples were compared from 15 SARS-CoV-2 naïve subjects. All were anti-N and anti-S negative before vaccination and only anti-S positive, 14 days after vaccination (Local Research and Development approval: CT1387/20).

### Analysis

Data files were uploaded to a secure portal to be accessed by the surveillance team at PHW. Staff from SBUHB and CTMUHB uploaded files of test results, each with a unique identifier and staff from WBS uploaded files containing donor demographic information and files containing lists that matched test results with demographic details. Staff from PHW then extracted the data from the portal and aggregated data at 4-week periods. Stata version 14 (StataCorp, College Station, United States) [11] was used for the analysis.

In order to provide a headline surveillance indicator, cell formulae in Microsoft Excel were used to calculate direct age-standardised seroprevalence using mid-year 2019 Welsh population estimates, by age group (17–29, 30–39, 40–49, 50–59, 60–69 and 70–85 years), which accounted for 79% of the population. This population structure was used to calculate the age-standardised value throughout. However, there were no donors in the 70–85-year-old group during period 1, likely due to national lockdown regulations.

Data were analysed by time, demography and location. For ethnicity, cumulative, as opposed to rolling seroprevalence was calculated, with black, Asian and minority ethnic groups being grouped as one due to low sample size. Lower Layer Super Output Areas were used to determine residential local authority (LA) and then LAs were grouped by the areas covered by local resilience fora: South Wales, Gwent, Dyfed and North Wales. Seroprevalence was presented as a percentage and was calculated by dividing the number of samples with a positive result for that period by the total number of samples included in the analysis for that period. Stata version 14 was used to calculate 95% binomial confidence intervals. Time trends of changes in seroprevalence by age group, sex and area of residence were plotted.

### Feedback of results

A short surveillance report was produced for each 4-week period, which was disseminated to key stakeholders involved in the pandemic response in Wales, such as the Health Minister for Wales.

### Additional analysis for this article

In samples that were tested for both anti-N and anti-S antibodies, the presence of both were quantified. Repeat donors were identified in order to investigate changes in positivity over time.

## Results

**Figure 1:**
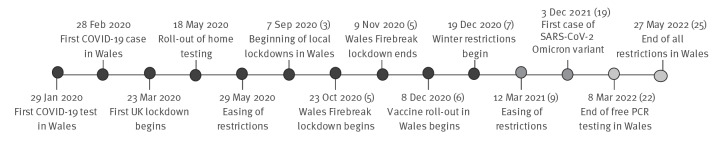
A timeline of key events during the COVID-19 pandemic, Wales, January 2020–May 2022

A total of 71,827 samples from 45,649 donors (female: 56.7% (25,900), male: 43.3% (19,749), median age: 46 years (33–57)) were tested and included in the analysis. A small proportion of samples (6.7%, 4,839 samples) were excluded due to the inability to link to demographic information, missing results or donors giving convalescent plasma. The latter were excluded as they were invited to donate as a result of a recent SARS-CoV-2 infection. Thirty-one 4-week periods were included with an average of 2,317 samples per period (range: 1,294–2,957). Figure 1 shows a timeline of key events throughout the pandemic, highlighting which period each event took place in.

Numbers in brackets indicate the 4-week period number.

### Seroprevalence over time

**Figure 2:**
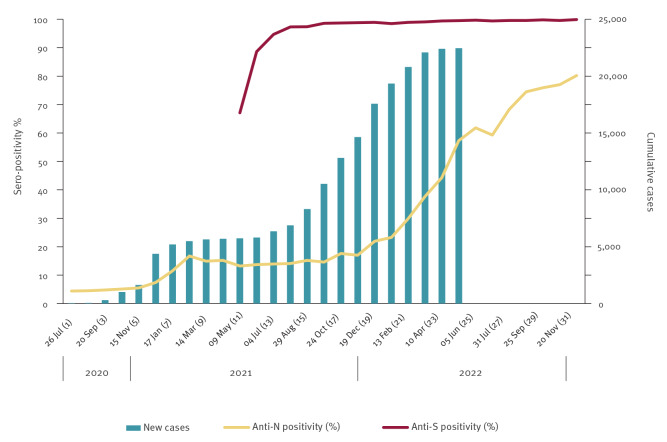
Seroprevalence of SARS-CoV-2 antibody in blood donors compared with the number of daily cumulative cases, based on the final day of each 4-week period, Wales, June 2020–November 2022

Anti-N: antibodies to the nucleocapsid antigen (resulting from natural infection); anti-S: antibodies to the spike antigen (natural infection and/or vaccine-mediated antibodies).

Periods 1–5 saw a relatively stable prevalence of anti-N antibodies (4.4%, 95% CI: 3.6–5.3 to 5.5%, 95% CI: 4.7–6.4 respectively). A rapid increase was then seen between period 6 (7.4%, 95% CI: 6.4–8.4), period 7 (11.5%, 95% CI: 10.2–13.0) and period 8 (16.7%, 95% CI: 15.1–18.4) before levelling out for the next 10 periods (period 18: 17.0%, 95% CI: 15.9–19.4). From this point, a steep upward trend was seen, peaking in the final period (period 31) (80.2%, 95% CI: 75.9–84.7). For anti-S antibodies, the direct age-standardised rate showed a marked increase from 67.1% (95% CI: 61.7–72.7) in period 11 to 98.6% (95% CI: 94.5–100) in period 16. A steady increase followed, reaching 99.1% (95% CI: 94.3–100) by period 22 and the prevalence continued to be above 99% for the remainder of the study (Figure 2).

Daily case reporting ceased in Wales 22 May 2022. The peak of infections in Wales was 21 December 2021 (16,252).

Data were aggregated over 4-week periods. Numbers in brackets refer to the corresponding 4-week period.

### Determinants of seropositivity

Males and females had an equal cumulative seroprevalence of anti-N antibodies (27.2%, 95% CI: 26.8–27.7), over the course of surveillance. This was also seen for anti-S antibodies (97.0%, 95% CI: 96.8–97.2).

**Figure 3:**
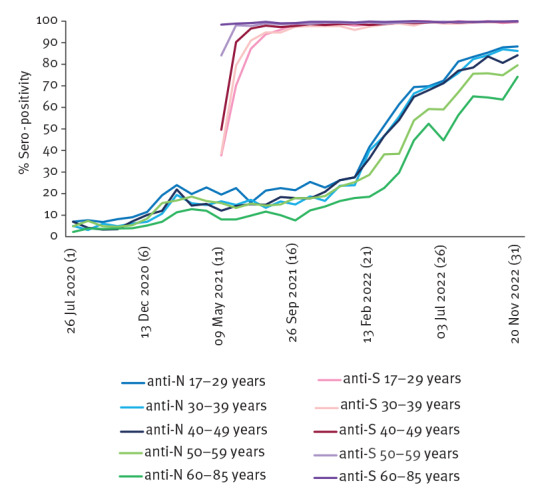
Age-specific prevalence of SARS-CoV-2 antibody (%) in blood donors, by antibody type and 4-week period, Wales, June 2020–November 2022

Anti-N: antibodies to the nucleocapsid antigen (resulting from natural infection); anti-S: antibodies to the spike antigen (natural infection and/or vaccine-mediated antibodies).

Significant differences in antibody prevalence (where confidence intervals (CI) do not overlap) were found between age groups. For anti-N antibodies, seropositivity remained highest in those aged 17–29 years throughout the study (cumulatively 30.8%, 95% CI: 30.0–31.7) compared to other groups aged 40–49 years (29.2%, 95% CI: 28.5–30.0); 30–39 years (28.9%, 95% CI: 28.1–29.7); 50–59 years (27.3%, 95% CI: 26.7–28.0); and 60–85 years (21.5%, 95% CI: 20.9–22.2). The highest prevalence of anti-S antibodies was seen in 60–85-year-olds (99.5%, 95% CI: 99.3–99.6) compared to age groups 50–59 years (98.6%, 95% CI: 98.4–98.8), 40–49 years (96.6%, 95% CI: 96.2–97.0), 30–39 years (94.5%, 95% CI: 93.9–95.0) and lastly 17–29 years (93.6%, 95% CI: 93.0–94.2). For both anti-N and anti-S antibodies, all age groups appeared to follow a similar pattern over time (Figure 3).

Data were aggregated over 4-week periods. Numbers in brackets refer to the corresponding 4-week period. Ethnicity has been recorded for approximately 93% of all blood donors since the beginning of 2020, with 2% reporting to be of Black, Asian or other minority ethnicity. Seroprevalence of anti-N antibodies was significantly higher in people self-reporting as Black, Asian or other minority ethnic group (34.0%, 95% CI: 31.3–36.7) than those self-reporting as White (27.0%, 95% CI: 26.6–27.3). Seroprevalence of anti-S antibodies was slightly higher in people of White ethnicity (97.0%, 95% CI: 96.9–97.2) than those of Black, Asian and other minority ethnicities (96.2%, 95% CI: 94.6–97.4).

Seropositivity of antibodies varied significantly depending on region of residence. Persons living in South Wales and Gwent had the highest cumulative rates of anti-N antibodies at 29.1% (95% CI: 28.6–29.6) and 28.0% (95% CI: 27.2–28.8), respectively. Anti-N antibodies rates were significantly lower in those living in North Wales (26.6%, 95% CI: 25.8–27.3 and Dyfed Powys (23.4%, 95% CI: 22.1–26.7).

**Figure 4:**
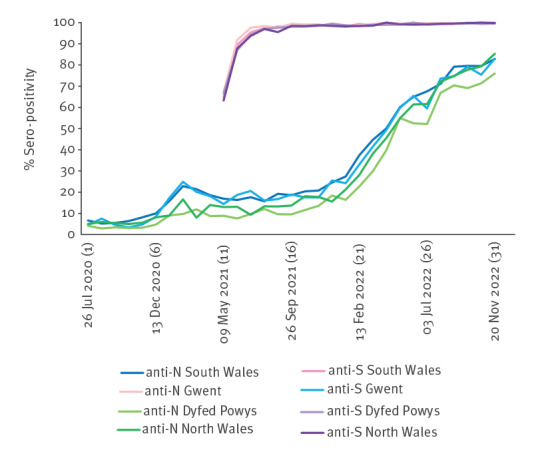
Resident location-specific prevalence of SARS-CoV-2 antibody (%) in blood donors, by type of antibody and 4-week period, Wales, June 2020–November 2022

Anti-N: antibodies to the nucleocapsid antigen (resulting from natural infection); anti-S: antibodies to the spike antigen (natural infection and/or vaccine-mediated antibodies).

For anti-S antibodies, the highest cumulative positivity was seen in Gwent (97.4%, 95% CI: 97.0–97.7), followed by South Wales (97.1%, 95% CI: 96.9–97.4), North Wales (96.8%, 95% CI: 96.4–97.2) and Dyfed Powys (96.7%, 95% CI: 96.3–97.0). Figure 4 shows the comparison of seropositivity by region of residence over time.

Data were aggregated over 4-week periods. Numbers in brackets refer to the corresponding 4-week period.

### Testing for anti-N and anti-S antibodies

**Figure 5:**
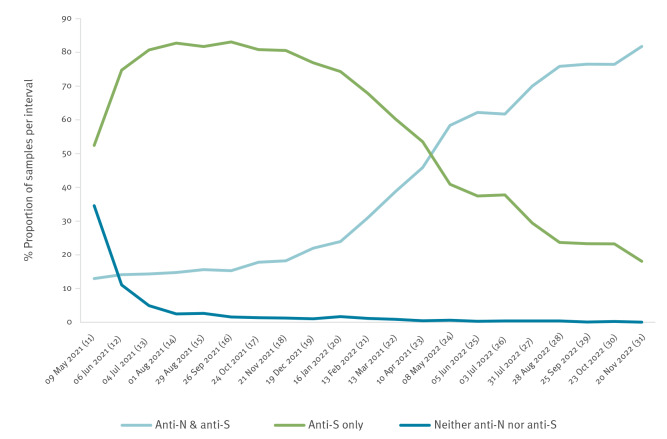
The proportion of donor blood samples with anti-N and anti-S antibodies by 4-week period, Wales, June 2020–November 2022

Anti-N: antibodies to the nucleocapsid antigen (resulting from natural infection); anti-S: antibodies to the spike antigen (natural infection and/or vaccine-mediated antibodies).

Between periods 11 and 31, 44,298 samples were tested for both anti-N and anti-S antibodies (Figure 5). A slight increase was seen in the proportion of samples that were positive for both antibodies, whereas there was a large increase in samples that were positive for anti-S antibodies but negative for anti-N antibodies. There was a decrease in samples that were negative for both anti-N and anti-S antibodies.

Data were aggregated over 4-week periods. Numbers in brackets refer to the corresponding 4-week period.

### Seroconversion and seroreversion

Due to repeat donations, 17,180 donors were tested between two and eight times for anti-N antibodies over the 31 periods. In addition, 9,003 donors were tested between two and six times for anti-S antibodies over the 21 periods. The results for repeat donor testing can be seen in the Table. For anti-N antibodies, a further 50 samples showed both seroconversion and seroreversion (and vice versa).

**Table:** Repeat donors tested for anti-N and anti-S antibodies by age, Wales, 29 June 2020–20 November 2022

Antibody	Donors								
	Age (years)	– at all donations		+ at all donations		Seroconverted (- to +)		Seroreverted (+ to -)	
		n	%	n	%	n	%	n	%
anti-N	17–29	1,189	13	362	19	844	15	25	22
	30–39	1,433	15	325	17	986	18	18	16
	40–49	1,712	18	392	20	1,185	21	29	25
	50–59	2,425	26	501	26	1,425	25	25	22
	60–85	2,730	29	340	18	1,167	21	17	15
anti-S	17–29	7	13	978	11	115	29	NA	
	30–39	20	36	1,194	14	125	32	NA	
	40–49	11	20	1,545	18	92	23	NA	
	50–59	10	18	2,350	27	51	13	< 5	100
	60–85	8	14	2,483	29	13	<5	NA	

−: negative result; +: positive result; anti-N: antibodies to the nucleocapsid antigen (resulting from natural infection); anti-S: antibodies to the spike antigen (natural infection and/or vaccine-mediated antibodies); NA: not applicable.

## Discussion

This serosurveillance system has successfully provided information about variation in seroprevalence of SARS-CoV-2 antibody in blood donors residing in Wales, by time, demography and location. Investigating both anti-N and anti-S antibodies to determine the cause of seroconversion corroborates data gathered through the monitoring of vaccine coverage and provides useful information on the distribution of susceptible individuals. Using a waste product from blood manufacturing is a low-cost method of acquiring blood samples.

Changes in antibody prevalence over time, illustrated by this study, are consistent with known variation in disease transmission and vaccination uptake in Wales. The initiation of the steep increase of anti-N antibodies corresponds with the emergence of the SARS-CoV-2 Omicron variant (Phylogenetic Assignment of Named Global Outbreak (Pango) lineage designation (B.1.1.529)) (27 November 2021 [12]), which was identified as more transmissible than the SARS-CoV-2 Wuhan strain that emerged in the UK in 2020 [13]. Repeatedly high prevalence of anti-S antibodies is consistent with high and continuous vaccine uptake in Wales [14].

Our key findings are generally in agreement with other reports of SARS-CoV-2 antibody within both blood donors and other populations. Young adults have frequently been found to have a higher rate of antibodies resulting from natural infection than their older counterparts, in many countries in Europe (e.g. Italy [15], Switzerland [16] and The Netherlands [17]). These findings are consistent with reports of increased transmission of COVID-19 among young adults [18,19]. Additionally, there is evidence that the rate of antibody decline has been found to be lowest in 18–24-year-olds and highest in those over the age of 70 years [20], supporting the increased presence of antibodies in younger individuals.

Globally, vaccine efforts were initially focused on older adults due to their higher rates of morbidity and mortality, therefore higher rates of anti-S antibodies are initially expected in older adults. This is consistent with the vaccine uptake figures reported by PHW [4]. Our findings suggest that the age-related gap between seropositivity may be due to the staggered eligibility for vaccination as positivity increased in other age groups over time.

Donors of self-reported Black, Asian and other minority ethnic backgrounds appeared to have a higher cumulative prevalence of antibodies resulting from natural infection than donors of White ethnicity. This is consistent with other findings [21,22] and reports of individuals being disproportionally affected by COVID-19 infections [23,24]. Donors of White ethnicity had a higher seroprevalence of anti-S antibodies than Black, Asian and other minority ethnic donors and this could support previous reports that vaccine hesitancy may be highest in some ethnic minority backgrounds [25,26].

For context, South Wales and Gwent regions cover urban South and South East Wales as well as the post-industrial Welsh Valleys. Dyfed Powys covers West and South West Wales and is typically rural with smaller population settlements. North Wales is both urban and rural, including the city of Wrexham, but also Wales’ largest national park, Eryri. South Wales and Gwent had a higher prevalence of anti-N antibodies than Dyfed Powys and North Wales. These significant variations in seroprevalence based on geographical location have been reported elsewhere [27] and other evidence indicates that outbreaks can even be localised to specific postcodes [28].

Our findings suggest that of all repeat donors, 55% tested negative for anti-N antibodies at all visits, suggesting that, although transmission rates have peaked at times, many individuals have avoided infection. Seroreversion rates appear to be low for anti-N antibodies, which is comparable to a study that found discordant results in 7% of people repeatedly tested for antibodies [21]. This is consistent with multiple reports that antibodies can last at least between 7 and 10 months [29-32] and that these findings were often limited by the length of the study. One instance of seroreversion of anti-S antibodies was found in our study which supports previous findings that anti-N antibodies wane more quickly than anti-S antibodies [33]. Therefore, anti-N antibodies can act as a proxy for re-infection within this cohort.

Free testing for the general population was discontinued in Wales in June 2022. This led to a reduction in testing and, consequentially, case numbers being identified through pre-existing surveillance systems. Donor-based serosurveillance provides continuous information about population immunity regarding SARS-CoV-2 post-pandemic, which has implications for future public health response, adding to the evidence base of how valuable the blood services facilitation of seroprevalence studies is [34,35]. Furthermore, potential for data linkage to public health databases in Wales, as done in Denmark [36], could be valuable for further interpreting the data.

Blood donors are not a fully representative sample of the population due to the inclusion criteria for donation and this impacts the generalisability of findings. A large percentage of donors lived in South Wales (43%). However, the majority of the population of Wales also live in South Wales (43% based on mid-2020 population data [37]). This suggests that the sample population’s residential demography is similar to the Welsh residential demography. Only 2% of samples were donated by people reporting Black, Asian and other minority ethnicity, which is less than the estimated 5.6% of the Welsh population (2020 [38]).

Interpretations of the repeat donor data are limited due to unknown infection and vaccination histories. It is possible that individuals could be re-infected between donations, thus appearing to have continued to hold antibodies when they have in fact seroreverted and seroconverted. Therefore, the true occurrence of seroreversion may not be being represented. This could be overcome by data linkage to other health databases.

## Conclusions

This surveillance system was successful in detecting changes in seroprevalence of antibodies to the nucleocapsid and spike antigens of SARS-CoV-2 over time. This information compliments other surveillance data on cases and vaccination uptake to provide an ongoing picture of the COVID-19 epidemic in Wales, informing its prevention and control through targeted testing and vaccination initiatives.
